# As to Bessie Wakefield

**Published:** 1914-01

**Authors:** 


As to Bessie Wakefield.—A woman by this name somewhere up in Connecticut killed her drunken brutal husband, and I say, served him right. But the jury with the facts before them thought differently, and sentenced her to be hanged March 4th. The brute had long previously kicked his little boy, making a cripple of him for life. There is a little daughter four years old. The case has attracted wide attention and raised a storm of protest all over the country, from men and women of all classes, who are shocked at the thought of hanging a *mother,* and who believe that the child will become an outcast, and, perhaps, in time the mother of a degenerate progeny. The protesters say "spare the mother and save the child, and prevent repetition of the Jukes record."

Well, the prospect of hanging the mother is very remote. I have it direct from the Governor of Connecticut, to whom I wrote, that the case is on appeal before the Criminal Court of the State; that if the sentence is affirmed the case will be appealed to the Supreme Court of the United States. If affirmed there, she has as last resort the Board of Pardons, of which the Governor is chairman. By that time I am pretty sure the Governor will be convinced that an advancing civilization will no longer tolerate the judicial murder of the mothers of children, and will turn her loose.

A great deal could be said on this subject, but I have no room, time or inclination to say it; further than if Bessie is pardoned, she should be sterilized, and the child should be taken out of an environment calculated to make an outcast of her.

